# Mitochondrial Dysfunction in Cardiac Diseases: Insights into Pathophysiology and Clinical Outcomes

**DOI:** 10.2174/011573403X379197250417061904

**Published:** 2025-05-06

**Authors:** Syed Shadab Ahmad, Javed Akhtar Ansari, Tarique Mahmood Ansari, Syed Mehdi Hasan Zaidi

**Affiliations:** 1 Faculty of Pharmacy, Integral University, Kursi Road, Lucknow, 226026, India

**Keywords:** Mitochondrial dysfunction, cardiac toxicity, cardiomyopathy, cardiovascular diseases, fatty acids, adenosine triphosphate

## Abstract

Mitochondrial dysfunction plays a crucial role in the pathogenesis of various cardiac diseases, including heart failure, ischemic cardiomyopathy, and drug-induced cardiotoxicity. Mitochondria are essential for cellular energy production, calcium homeostasis, redox balance, and apoptotic regulation, making their proper function vital for cardiac health. Dysfunctional mitochondria contribute to excessive reactive oxygen species (ROS) production, impaired ATP synthesis, and disruption of mitochondrial dynamics, leading to cardiomyocyte damage and cell death. Emerging research highlights mitochondrial dynamics, including fission, fusion, mitophagy, and biogenesis, as critical determinants of cardiac homeostasis. Perturbations in these processes exacerbate myocardial injury and heart failure progression. Additionally, chemotherapy-induced cardiotoxicity, primarily from anthracyclines, is closely linked to mitochondrial damage, underscoring the need for targeted therapeutic strategies. Pharmacological interventions, such as antioxidants, mitochondrial-targeted drugs, and cardioprotective agents, have shown promise in mitigating mitochondrial dysfunction-related cardiac toxicity. Furthermore, lifestyle modifications, including exercise and dietary interventions, are being explored to enhance mitochondrial resilience in cardiac tissues. Advanced imaging techniques and biomarker-based diagnostics are improving the early detection of mitochondrial dysfunction in cardiac diseases. Emerging therapeutic strategies, such as mitochondrial transplantation, gene therapy, and precision medicine approaches, hold potential for targeted intervention. Despite these advances, challenges remain in translating mitochondrial-targeted therapies into clinical practice due to complexities in mitochondrial regulation and inter-organ communication. Future research should focus on optimizing mitochondrial-targeted interventions, improving diagnostic precision, and exploring novel molecular pathways to mitigate cardiac mitochondrial dysfunction. A comprehensive understanding of mitochondrial pathophysiology in cardiac diseases will pave the way for innovative treatment strategies aimed at preserving cardiac function and reducing the burden of heart failure.

## 1 INTRODUCTION

Mitochondria are vital organelles found in almost all eukaryotic cells. Mitochondria, often referred to as “the powerhouse of the cell”, play an essential role in providing adenosine triphosphate (ATP) by oxidative phosphorylation (OXPHOS), a biochemical pathway that converts nutrients into high-energy phosphate molecules, which provide energy to sustain most cellular and physiologic processes [1]. Although most tissues can use either carbohydrate or lipid as energy substrates, cardiac myocytes prefer (FAs), derived from lipolysis by the action of cardiac lipases and by the uptake of circulating low-density lipoproteins/very-low-density lipoproteins transported by specific receptors. Once at the mitochondrial inner membrane, FAs undergo a series of metabolic transformations known as β-oxidation, leading to the formation of acetyl coenzyme A (acetyl-CoA). Mitochondria are also instrumental in the maintenance of the intracellular pH and calcium homeostasis, in lipid and heme biosynthesis, in the regulation of the cellular redox state, and apoptotic cell death [2]. To accomplish their activities, mitochondria exploit a selective quality control machinery to target and remove misfolded proteins or aberrant organelles. Mitochondria are involved in the modulation of nuclear gene expression that may regulate crucial pathways involved in cell survival. Mitochondrial impairment has been implicated in diverse pathological conditions, including ischemic cardiomyopathy, heart failure, stroke, cardiac hypertrophy, and diabetes. Mitochondria are dynamic organelles capable of rapid and continuous changes in shape, size, number, and location by biogenesis, fusion, fission, degradation, and motility, contributing to cellular homeostasis and adapting to ever-changing physiological demands [3-5].

### 1.1 Structure and Function of Mitochondria in Cardiomyocytes

Mitochondria are double-membrane organelles distributed throughout the cytoplasm and are integral to crucial cellular processes, including energy, biosynthesis, metabolism, and cell death. Mitochondrial dysfunction is associated with a wide range of diseases, such as immigration syndrome (MIAS), Leigh syndrome (LS), and neurodegenerative and cardiovascular diseases [6]. Mitochondria are the major sources of energy for cardiomyocytes (CMs) and play a central role in overall cardiac function, including calcium homeostasis, cellular signaling, and oxidative stress. Because of these significant roles, mitochondrial dysfunction alters metabolism and energy production which may lead to cardiac toxicity [2]. The complex structure enables high efficiency in generating adenosine triphosphate (ATP) *via* oxidative phosphorylation (OXPHOS) through the electron transport chain (ETC). It also permits the formation of a strong electrochemical proton gradient across the inner mitochondrial membrane (IMM), harnessed both to drive ATP production and regulate the spatial and temporal distribution of several ions and metabolites, such as calcium ions (Ca^2+^), reactive oxygen species (ROS), ammonium bases, and lipids. Transmission electron microscopy (TEM) studies on healthy hearts revealed the presence of different mitochondrial morphologies, including rounded or rod shapes, elongated forms, and small clusters surrounding the nucleus and within the myofibrils. The complex three-dimensional (3D) structure of mitochondria depends on the balance between biogenesis and dynamics processes regulated by mitochondrial fission, fusion, and degradation, which in turn, may impact cellular events, such as calcium homeostasis, metabolism, and apoptosis [7-9]. Fig. (**1**) illustrates the structural components and functional roles of mitochondria. The diagram highlights the outer and inner membranes, intermembrane space, and matrix as key structural features, along with their respective functions, such as ATP production *via* the electron transport chain, fatty acid metabolism (β-oxidation), and the citric acid cycle. Additionally, the figure emphasizes mitochondria's critical functions, including calcium homeostasis, regulation of reactive oxygen species (ROS), and apoptosis.

### 1.2 Importance of Mitochondria in Cardiac Health

Mitochondria are the vital organelles that actively participate in the regulation of cellular metabolism and bioenergetics, cytosolic and mitochondrial calcium ion homeostasis, cell death, and production of reactive oxygen species (ROS); hence, they help in maintaining cell health [10]. The heart relies on mitochondria for the majority of its energy production and a plethora of other functions that are important for maintaining cardiac health. Mitochondria-related cardiac toxicity may lead to arrhythmias, energy deficiency, necrotic death, and/or apoptosis, which are critical in the mechanism of cardiac diseases. The pharmacological and environmental cardio-toxicants may lead to mitochondrial dysfunction, which can affect cardiac bioenergetics and signal pathways involved in a myriad of cardiac alterations and pathologies [2].

Mitochondria are the main source of ATP production under aerobic conditions by oxidative phosphorylation, which is necessary for normal cell function and viability. Because of the high energy requirement of cardiac tissue, mitochondria account for approximately 30% of cell volume and generate most of the energy needed for the heart. Mitochondria are intimately involved in metabolic signaling integrated from the extra- and intracellular milieu and coordination of ancillary mechanisms such as ROS production, transport of Ca^2+^ ions, thermogenesis, and programmed cell death [11, 12].

## 2 CARDIAC TOXICITY: DEFINITION AND TYPES

Cardiotoxicity refers to cardiac damage caused by the excessive accumulation of endogenous or exogenous substances to reach a toxic concentration. During the toxic concentration of these substances, cardiac rhythm or cardiac electrical activity changes occur. Cardiotoxicity can cause cardiac electrophysiological dysfunction or myocardial damage, which in turn leads to heart failure, arrhythmia, and/or sudden cardiac death [2].

With the development of high-throughput screening technology and enhanced traditional drug efficacy screening models, especially drug efficacy screening models dealing with cardiotoxicity, the early assessment of drug efficacy can be realized. With the progressive development of chemical biology and biophysics techniques, many small molecules or chemical inhibitors that perturb assorted molecules or cellular signaling have been discovered. These small molecules or inhibitors, however, might involve multiple targets due to the complicated cellular pathways. This non-specificity raises questions about the safety of drugs with high toxicity [13-15]. While a part of drugs is specified to a target, their onset might occur after usually long exposure time, or a companion toxicity displays at the same dose or blood concentration. Such a situation happens repeatedly for newly approved drugs in cancer chemotherapy, anti-virus infection, and new anesthetics, as well as for clinically used molecular probes for biological research or new drugs under pre-clinical assessment for safety. Thus, the recent clean-up and enforcement of toxicity assessment point out the demand for high-throughput toxicity assessment models, along with clear mechanisms of toxicity.

While toxicity might occur to organs that contain targets accidentally, cardiotoxicity has been recognized to be caused by the intentional or accessory action of drugs with high affinity to heart-related targets or pathways. For instance, a drug designed to upset intracellular calcium homeostasis or to inhibit energy metabolism, thus influencing heart rhythmic contraction, may lead to an overt cardiac toxicity. Thus, high-throughput screening platforms capable of easily modeling heart-related toxicity and studying the involved mechanism in either overt or sub-lethal levels are of great importance. More attention and efforts are still needed to both complementary technique discovery and safety knowledge deposition to protect people from drug toxicity while enjoying the benefit of drug-improved life [16-18].

### 2.1 Chemotherapy-induced Cardiotoxicity

Cardiotoxicity encompasses the unwanted side effects on heart tissue post-exposure to certain drugs, leading to adverse cardiac events following therapy. There exists a diversity of agents related to both cancer and non-cancer medications that can exert cardiac toxicity. Cardiotoxicity resulting from chemotherapeutics is known as chemotherapy-induced cardiotoxicity (CIC). It is gradually recognized that several agents, previously viewed as unlikely culprits due to the absence of cellular toxicity in normal tissues, have the potential to induce cardiotoxicity. Among the largest class of antitumor medications with well-documented cardiotoxic effects are anthracyclines [19-21]. Doxorubicin was the first and most widely used drug of this family and has been indicated for the treatment of breast, bladder, lung, leukemia, and soft tissue cancers. Depsipeptide, also known as romidepsin (FK228), is a natural product derived from the culture broth of a soil bacterium. This compound entered phase I of clinical trials, demonstrating rules of engagement concerning safety and tolerability in humans. Sensitivity to depsipeptide toxicity may exist in individuals, ultimately leading to undesired cardiac effects with potential implications in drug dosage [22].

Anthracyclines are amongst the most potent anticancer drugs employed in the treatment of multiple human malignancies, including leukemia, lymphomas, and a wide spectrum of solid tumors in breast, lung, head-and-neck, and soft tissues. Herein, explorations are reported linking mitochondria to the pathophysiology and clinical outcomes of CIC as a consequence of acute toxic events. Ongoing efforts have been devoted to understanding the cellular and molecular mechanisms responsible for cardiotoxic effects of anticancer chemotherapeutic drugs. Despite the disclosure of various cellular events involved in CItox, mitochondria remain underexamined in the pathology of a cancer-induced heart suffering during and after chemotherapy. Before cellular reprogramming towards hyperproliferation and apoptosis escape, one mechanism of CItox is suggested to stem from mitochondrial perturbations such as energetic failure, ROS overproduction, or phospholipid desaturation [23-25]. To illustrate the range of chemotherapy drugs associated with cardiotoxic effects, Table **1** provides a summary of key agents, their mechanisms of action, and their impact on mitochondrial and cardiac health.

### 2.2 Drug-induced Mitochondrial Dysfunction

Mitochondrial dysfunction has been increasingly recognized as a critical mechanism underlying cardiac toxicity induced by various drugs. The heart is a highly energy-demanding organ that relies heavily on oxidative phosphorylation (OXPHOS) for adenosine triphosphate (ATP) production. Mitochondria are crucial organelles for ATP generation, on which cardiomyocyte contractility depends. Mitochondrial impairment, including both structural and functional alterations, leads to the development of cardiovascular diseases (CVDs). Large numbers of mitochondrial dysfunction-related CVDs have been discovered, including atherosclerosis, ischemic heart disease, heart failure, and atrial fibrillation [2]. Chemotherapeutic agents, antibiotics, and antipsychotic drugs can induce cardiac mitochondrial dysfunction and contribute to the pathogenesis of drug-induced cardiotoxicity. Mitochondrial Ca^2+^ overload, excessive oxidative stress, mitochondrial permeability transition pore (mPTP) opening, and mitophagy dysregulation have been implicated in cardiac toxicity. Mitochondrial dysfunction is closely associated with clinical outcomes, such as the development and severity of drug intervention-related cardiac toxicity. Mitochondrial mechanisms can be targeted to develop potential treatment strategies and reduce the risk of cardiac toxicity in patients at high risk or undergoing treatment with cardiotoxic drugs [1]. Mitochondria perform multiple critical functions for a cell, the most well-known being the production of ATP by oxidative phosphorylation (OXPHOS). The impairment of these organelles can drastically affect cardiac homeostasis, promoting the development of cardiovascular diseases (CVDs). Anthracyclines are widely used chemotherapeutic drugs with a well-characterized mitochondrial cardiotoxicity. Additionally, the off-target effects of CNS-acting drugs and antibiotics can also affect mitochondrial function, enhancing their cardiotoxicity. Mitochondrial dysfunction is a common denominator of most CVDs, regardless of their origins [32, 33]. It may result from various causes, including genomic and mitochondrial DNA (mtDNA) mutations, oxidative stress, accumulation of pathological proteins, calcium overload, and diabetes-induced disturbances. Mitochondrial dysfunction is often associated with the activation of the mitochondrial apoptosis pathway, leading to cardiomyocyte cell death. In severe conditions, as in the context of ischemia, the progressive dysfunction of mitochondria and Ca^2+^ overload can also promote necrotic cell death [34]. Fig. (**2**) illustrates the mechanisms through which drug exposure induces mitochondrial dysfunction and subsequent cardiac toxicity. It highlights two primary pathways: direct mitochondrial damage, resulting in structural alterations and DNA depletion, and increased reactive oxygen species (ROS) production, leading to oxidative stress. These pathways converge to cause mitochondrial membrane damage, decreased ATP production, and activation of mitochondrial permeability transition pores (mPTP), ultimately resulting in ion imbalance, apoptosis, and cardiac toxicity.

## 3 MITOCHONDRIAL DYSFUNCTION IN CARDIAC PATHOPHYSIOLOGY

Cardiac dysfunction is a major complication in patients with heart diseases, especially diabetes mellitus (DM). Mitochondria, double-membrane organelles, play key roles in various cellular functions, including oxidative phosphorylation (ATP production), regulation of reactive oxygen species (ROS), and calcium homeostasis. Increasing evidence demonstrates that mitochondrial dysfunction significantly contributes to the pathophysiology of several heart diseases. For instance, heart rhythm disorders such as atrial fibrillation, cardiac arrest, and sudden death can occur as early events in heart disease progression, with mitochondrial dysfunction accelerating these conditions [35].

Mitochondrial dynamics, which involve fission, fusion, trafficking, and degradation, are crucial to maintaining cellular health. Advances in imaging technologies, such as fluorescence probes and high-content imaging, now enable real-time analysis of mitochondrial processes like fission, fusion, and mitophagy. Alterations in these processes, such as excessive fission, impaired fusion, or abnormal mitophagy, can lead to various pathological conditions that disrupt cellular homeostasis [1]. Abnormal mitochondrial dynamics are now recognized as a significant factor in cardiac pathologies, especially in conditions associated with heart dysfunction. For example, mitochondrial dysfunction is a key contributor to cardiac toxicity induced by anti-cancer drugs [36, 37]. A detailed overview of mitochondrial dysfunction markers relevant to cardiac pathophysiology is presented in Table **2**, highlighting their roles, detection methods, and links to cardiac toxicity.

Fig. (**3**) illustrates the dual role of the mitochondrial electron transport chain (ETC) in cellular metabolism and redox balance. The ETC facilitates proton pumping across the mitochondrial membrane, creating a proton gradient that drives ATP production *via* ATP synthase. Concurrently, the ETC generates reactive oxygen species (ROS) as a byproduct. The balance of ROS levels is critical for cellular health. While moderate ROS levels are essential for signaling and maintaining cellular function, excessive ROS leads to oxidative stress, which is associated with pathological conditions. This schematic emphasizes the delicate balance required between energy production and ROS regulation for maintaining cellular homeostasis.

### 3.1 Role of Mitochondria in Energy Production

Mitochondria are dynamic organelles primarily distributed in the cytoplasm, with two key compartments: the outer mitochondrial membrane (OMM) and the inner mitochondrial membrane (IMM), separated by the intermembrane space (IM). They are central to energy metabolism, cell signaling, calcium storage, and apoptosis. ATP synthesis occurs *via* oxidative phosphorylation, where ATP synthase, located on the IMM, couples proton translocation across the inner membrane with ATP production.

Mitochondria also regulate cellular redox balance, producing ROS, which, in moderate amounts, are essential for normal cellular functions such as redox signaling. However, excessive ROS production, often caused by mitochondrial dysfunction, overwhelms antioxidant defenses and leads to oxidative stress. This contributes to cellular damage and dysfunction, playing a significant role in the pathogenesis of heart diseases, as well as other conditions like neurodegenerative and metabolic diseases [44-47].

Cardiotoxicity refers to damage caused by toxic concentrations of endogenous or exogenous substances, leading to electrophysiological dysfunction or myocardial injury. Mitochondria are central in maintaining energy metabolism, redox homeostasis, and cell survival. Mitochondrial dysfunction is often the initial step in cardiotoxicity. Exogenous substances like chemotherapeutic drugs, environmental pollutants, and alcohol can disrupt mitochondrial function, predisposing cells to toxicity. Cardiac disturbances resulting from these substances can occur at the level of excitation-contraction coupling, leading to rhythm abnormalities, calcium handling defects, and cell death processes such as necrosis and apoptosis [1, 48-51].

### 3.2 Reactive Oxygen Species and Oxidative Stress

Mitochondria are the main source of reactive oxygen species (ROS) inside the cardiac cells. Under physiological conditions, ROS play a pivotal role in maintaining cardiac functions by modulating redox-sensitive signaling pathways and cellular processes. An overproduction of ROS, however, can result in oxidative stress, leading to oxidized macromolecules and dysfunction in cellular structure and signaling. Oxidative stress is recognized as an important mechanism accounting for a wide spectrum of cardiac pathophysiology, including I/R injury, cardiomyopathy, diabetic cardiomyopathy, heart failure, and so forth [2]. Accumulating evidence shows that anticancer agents can induce oxidative stress by modulating electron transfer chain complexes in mitochondria, ultimately resulting in cardiac toxicity [1].

## 4 MOLECULAR MECHANISMS OF MITOCHONDRIA-RELATED CARDIAC TOXICITY

Mitochondria are the crucial energy-producing organelles in cells and are responsible for cellular energy and metabolism. It has been reported that mitochondrial impairment is associated with various diseases, such as cancers, renal dysfunctions, and cardiac dysfunctions. Cardiac injury can occur due to various reasons, such as chemotherapy treatment, hypertension, myocardial ischemia, and myocardial infarction. Although the initial reasons vary, pathological changes of various cardiac injuries ultimately converge on mitochondrial injury. There is increasing evidence that heart mitochondria play essential roles in cardiac toxicity. Mitochondria-related cardiotoxicity refers to cardiac damage caused by the excessive accumulation of substances in cells to a toxic concentration in heart mitochondria, leading to harmful effects on cardiac physiological functions. When cardiac cells are stimulated by pathogenic factors, such as reactive oxygen species (ROS), changes in the shape, structure, and function of mitochondria occur. Excessive stress causes mitochondrial energy metabolism and quality control to be disturbed [52].

Mitochondrial dynamics and mitophagy are crucial for the maintenance of normal mitochondrial numbers, shape, and function, and, under stress conditions, to reshape and remove dysfunctional mitochondria [1]. Pathogenic factors activate multiple signaling pathways, promoting mitochondrial fission or fusion. Altered engagement of mitochondrial dynamics results in cardiac toxicity. Accumulative evidence indicates that mitochondrial-related cardiotoxicity is associated with cardiac dysfunction, myocardial cell viability loss, alteration in bioenergetics, and disruption of calcium homeostasis [53-55]. Moreover, the response of heart mitochondria to pathogenic factors involved in the onset and progression of cardiac injury diseases, including cardiac hypertrophy, ischemia-reperfusion injury, and doxorubicin-induced cardiotoxicity, has been reviewed. Accumulative evidence indicates that the alteration of mitochondrial dynamics and mitophagy plays important roles in the initiation and maturation of mitochondrial toxicity, which is associated with cardiotoxicity [56, 57]. Fig. (**4**) illustrates the sequence of events involved in mitochondrial cardiac toxicity. The process begins with mitochondrial DNA damage, primarily induced by reactive oxygen species (ROS), which triggers cellular repair mechanisms. If these repair efforts fail or are insufficient, the mitochondrial permeability transition pore (mPTP) opens, disrupting mitochondrial function. This leads to mitochondrial swelling and the eventual loss of mitochondrial membrane potential. The culmination of these events is cell death, driven by profound mitochondrial dysfunction. This pathway underscores the critical role of mitochondrial integrity in maintaining cardiac cellular homeostasis.

### 4.1 Mitochondrial DNA Damage and Repair

Mitochondria, known as the powerhouses of the cell, are dynamic organelles that produce energy for cell processes by generating ATP *via* oxidative phosphorylation in mammals. They are also involved in other cellular functions, such as calcium homeostasis, apoptosis, and cellular metabolism regulation. Beyond the nucleus, mitochondria possess their own circular double-stranded mtDNA, which is 16.5 kb long in humans [58]. Unlike nuclear DNA, mtDNA is transcriptionally coupled with OXPHOS complexes. It is also more susceptible to various stress-induced damage due to its unique characteristics. Therefore, a battery of mtDNA maintenance and repair processes occurs within the mitochondria to ensure mitochondrial function and cellular bioenergetics [59]. Excessive mtDNA damage, if not repaired efficiently, may lead to mitochondrial dysfunction and a variety of diseases related to bioenergetic defects (*e.g.*, neurodegenerative diseases, muscular dystrophies, diabetes, aging, and cardiovascular phenotypes) [1].

In mammals, despite the pathogenic outcomes, insight into the mechanisms of mtDNA damage and repair is still very much needed before any consideration of therapeutic applications. Thus far, five DNA repair mechanisms have been identified in mammalian mitochondria, which include 1) the base excision repair (BER), 2) the single strand break repair (SSBR), 3) the homologous recombination repair (HRR), 4) the nonhomologous end joining repair (NHEJR), and 5) mitochondrial DNA degradation pathway. Mechanistically, five categorized mtDNA damage events have been identified, which include 1) oxidative base damage, 2) simple and complex single-strand breaks, 3) double-strand breaks, 4) DNA polymerase stalling, disrupted replication fork, and 5) intermediate generation in the degradation pathway [60, 61]. These events result in the accumulation of damaged mtDNA, which can manifest as multiple 1-kb long deletions in the mtDNA sequence and ultimately mtDNA depletion. Systematic understanding of the basic mechanisms of the repair pathways is required to divulge their cellular and pathogenic consequences [62, 63]. The types of mitochondrial DNA damage, associated repair mechanisms, and their implications for cardiac health are summarized in Table **3**, offering insights into this critical aspect of cardiac toxicity.

### 4.2 Mitochondrial Permeability Transition Pore

Mitochondrial permeability transition pore (MPTP) is a multi-subunit complex that mediates a non-selective, high-conductance permeability of the mitochondrial inner membrane to solutes of less than 1.5 kDa after a critical threshold is exceeded for various trigger factors [69]. The initial aim of MPTP studies focused on a better understanding of how Ca^2+^ overload and oxidative stress lead to energy depletion and cell death. However, this area of research now attracts the attention of a wider community of scientists. Exciting developments help to understand the identification of MPTP molecular components and the mechanistic details surrounding the modulation of MPTP activity by pathophysiological signals. Patients with binge alcohol drinking have impaired ability to develop myocardial protection against an ischemia-reperfusion insult, which can be related to an 8-fold upregulation of mitochondrial pore opening (mPTP). This observation sheds further light upon the role of mPTP in adaptation to ischemia and lays the groundwork for developing a fresh paradigm for the development and implementation of clinically viable pharmacological interventions. It confirms mPTP as a robust mechanism to mediate mitochondrial dysfunction and cell death after a variety of toxic stimuli. Accumulating evidence demonstrates that both cardiac and non-cardiac right ventricular (RV) pacing, if implemented aggressively, transiently induce mild and adaptive mitochondrial dysfunction prior to promoting reversible contractile dysfunction. There are emerging concerns regarding the effects of mitochondrial dysfunction on cell health and the development of air pollution-related cardiac toxicity. Advances in knowledge of several mitochondrial processes can now be harnessed to develop new therapeutic strategies to combat acute-to-chronic cardiac toxicity [70-73].

## 5 CLINICAL MANIFESTATIONS OF MITOCHONDRIA-RELATED CARDIAC TOXICITY

Mitochondria-related cardiac toxicity ultimately leads to various acute and chronic cardiac diseases, manifesting as heart failure or cardiomyopathy, arrhythmias, sudden cardiac death (arrhythmic), ischemic heart disease (angina), and perioperative cardiac events (tissue hypoperfusion). Mitochondrial oxidative stress, excessive mitochondrial fission, and defective mitochondrial autophagy are the critical pathogenic pathways for various toxic cardiac injuries. Recently developed protective strategies targeting mitochondrial dynamics, mitophagy, bioenergetic metabolism, and redox homeostasis would provide new, timely therapeutic or preventive options against mitochondria-related cardiac toxicity [74].

The heart, a muscular organ about the size of a fist, beats about 100,000 times a day and pumps 2,000 gallons of blood throughout the body. The constant pumping motion is generated and maintained by normal electrical activity and mechanical contraction in cardiac myocytes or cardiomyocytes, the functional unit of the heart. Cardiac cells, however, are often exposed to various internal or external physico-chemical stresses (danger signals), which can cause cardiac electrophysiological or mechanical dysfunction in pathophysiological states. Mitochondria, the powerhouse of eukaryotic cells, provide almost all the energetic ATP for sustaining physiological activity, biosynthesis, and repair in cells [2]. However, when cardiac cells are stimulated by internal or external danger signals, the overload accompanies various physiological responses, including disruption of mitochondrial dynamics and intracellular iron metabolism, which are thought to be the common pathophysiological events for various toxic cardiac injuries [75]. Fig. (**5**) provides an overview of the symptoms and diagnostic techniques associated with cardiac conditions. Common symptoms include fatigue, shortness of breath, chest pain, palpitations, syncope, and exercise intolerance. To assess these symptoms, various diagnostic methods are employed. Biomarkers, such as cardiac troponins and B-type natriuretic peptide (BNP), are used for biochemical analysis. Echocardiography, including transthoracic echocardiography (TTE) and Doppler studies, offers imaging insights into cardiac function. Advanced imaging modalities, such as cardiac MRI and positron emission tomography (PET), further aid in detailed structural and functional assessments. This framework highlights the integrative approach to diagnosing cardiac diseases.

### 5.1 Symptoms and Diagnostic Approaches

Many of the tests and approaches commonly deployed to assess cardiac health are impossible in the case of cardiac toxicity that may stem from mitochondrial dysfunction. Common diagnostic tests include creatine kinase blood test, myeloperoxidase blood test, Natriuretic Peptides blood test, and troponin blood test, which are all indirect, instead looking at the cascade of other consequences of cardiac toxicity rather than simply measuring mitochondrial function directly [1].

### 5.2 Imaging Modalities for Cardiac Dysfunction

MitoTox can occur long after the initiating causative event, spanning years to decades. Given the variety of pro-cardiotoxic agents, endpoints, and timelines associated with MitoTox, a variety of approaches have been employed to assess cardiac toxicity in preclinical species and the clinic. At an organ level, imaging modalities such as echocardiography, cardiac magnetic resonance imaging (MRI), and nuclear imaging have provided insights into cardiac toxicity associated with chemotherapeutics and targeting an array of cardiac pathophysiologies, including those relating to cardiomyopathy [76]. Table **4** outlines diagnostic approaches for identifying mitochondria-related cardiac dysfunction, emphasizing techniques, target biomarkers, and their relative strengths and limitations.

## 6 CURRENT TREATMENT STRATEGIES FOR MITOCHONDRIA-RELATED CARDIAC TOXICITY

Mitophagy is receiving considerable attention as a protective mechanism against mitochondrial dysfunction and related diseases. Inducers of mitophagy, including either overexpression of components required for mitophagy or inhibition of components preventing mitophagy, are reported to have protective effects against mitochondrial damage [83]. However, a comprehensive exploration of potential new agents, especially natural products, to induce mitophagy to reduce mitochondrial damage, inflammation, apoptotic changes, and DNA damage during cadmium exposure and to elucidate the underlying mechanisms in murine cardiomyoblast cells is necessary. Mitochondrial impairment has been identified as a key structural and functional alteration in mitochondria that leads to contractile dysfunction and underlies the pathophysiology of several cardiovascular diseases. Mitochondrial dysfunction is implicated in the pathogenesis of heart failure with preserved ejection fraction (HFpEF). Mitochondria are also thought to be key channels of cell death in heart disorders. They can directly induce cell lysis by rupture of the mitochondrial outer membrane, mitochondrial permeability transition pore (mPTP) opening, and release of pro-apoptotic factors. Recent evidence has also uncovered a connection between mitochondrial dysfunction and inflammatory/immune responses in the heart, suggesting an involvement of mitochondria in the regulation of inflammation and autoimmunity [84-86]. The therapeutic interventions targeting mitochondria-related cardiac toxicity are summarized in Table **5**, which includes pharmacological options and lifestyle strategies, their mechanisms, and effectiveness.

Fig. (**6**) outlines various approaches to mitigate mitochondria-related cardiac toxicity. These include pharmacological interventions such as antioxidants, mitochondrial protectants, and heart failure medications; lifestyle modifications like dietary changes, exercise, and stress management; and emerging strategies such as exercise-induced mitochondrial biogenesis, targeted gene delivery, pharmacological agents, and Clustered Regularly Interspaced Short Palindromic Repeats (CRISPR)-based gene therapy. This comprehensive framework highlights the potential for both established and innovative methods to address mitochondrial dysfunction in cardiac health.

### 6.1 Pharmacological Interventions

Pharmacological interventions are key approaches in the management of mitochondrial-related cardiac toxicity, which is a common and serious complication of cytotoxic anti-neoplastic therapeutic agents. Most drugs, which were previously found to induce cardiotoxicity, are either anti-neoplastic agents like anthracyclines (doxorubicin), proteasome inhibitors, anti-vascular agents, tyrosine kinase inhibitors, and antitumor antibiotics (damoxetone), or non-cytotoxic agents like alcohol, lithium salts, azathioprine, and others [93-95]. These drugs can attack the normal and healthy cells and tissues in the heart, and the accompanying damage to cellular and organ structures leads to the development of heart failure (HF). Salient examples of cardiac toxicities induced by mitochondrial dysfunction machinery and lysosomal blockage of bioenergetic impairment include doxorubicin cardiotoxicity, proteasome inhibitor (bortezomib and carfilzomib) cardiotoxicity, and cardiac toxicity of anthracycline antibiotics (mitoxantrone, dactinomycin, and bleomycin), apologues [96, 97].

Protection against drug-induced cardiac toxicity has been attempted by multiple ameliorative strategies. Some of these may be considered as preventive measures and are based on the application of drugs influencing the organ distribution and bioactivation of the toxic agent, either limiting retention in cardiac tissues or enhancing bio-inactivation. In the research arena, pharmacological agents influencing cellular processes like antioxidant endothelin receptor blockers are beginning to be tested as possible ways for ameliorating the myocardial side effects of anticancer drugs [26, 98, 99]. A meticulous search and study of experimental literature reveal that several of these agents, properly applied, have beneficial effects against mitochondrial-related cardiac toxicity and ameliorative pharmacological options. Targeting mitochondrial machinery has beneficial effects on anthracycline-induced cardiotoxicity, and therefore attenuates doxorubicin-induced cardiotoxicity by activating various signaling cascades and modulating pro-apoptotic and anti-apoptotic proteins [100].

### 6.2 Lifestyle Modifications and Dietary Approaches

Previous studies [1, 2] have implicated the potential role of exercise and lifestyle modifications in ameliorating mitochondrial dysfunction in numerous disease conditions, including heart disease and aging. These experimental findings suggest that exercise could have protective effects against cardiac toxicity associated with mitochondrial dysfunction and could serve as a preventive strategy in patients taking drugs that are known to affect cardiac mitochondrial health. These studies point out several lifestyle modifications that, in addition to exercise, could also bolster mitochondrial health and limit the risk of development or worsening of mitochondrial toxicity. For example, could dietary approaches using food or supplements rich in anti-oxidant nutrients be an adjuvant therapy based on the beneficial effect of exercise [101-104]?

Looking specifically at some of the nutritional approaches that could be employed, a high-carb, low-fat, *e.g.*, ketogenic, diet has been found to restrict mitochondrial dysfunction and the accompanying activation of pathways thought to contribute to toxicity. Ketogenic diets improve mitochondrial bioenergetics and ROS management by enhancing complex I, II respiration and mitochondrial membrane potential and reducing their aberrant activation. Such diets also curtail the expression of proteins involved in ROS signaling pathways, proteotoxicity, and mitochondrial calcium overload [105, 106]. In addition, ketone bodies could serve as an alternative fuel for cardiac metabolism, as evidence suggests that ketogenic diets, in exercised-trained individuals, could be associated with improved myocardial oxygen consumption and efficiency, particularly in settings of mitochondrial dysfunction. However, caution should be exercised, as certain individuals in the absence of proper neurological assessment could transiently experience side effects originating from a strict high-fat low-carb entry into the diet [107-109].

## 7 EMERGING THERAPEUTIC TARGETS AND FUTURE DIRECTIONS

The growing focus on mitochondrial-related cardiac toxicity, particularly in the context of cardiotoxicity induced by anticancer therapeutics, has led to the identification of critical factors contributing to the onset and progression of cardiac diseases. These include mitochondrial dynamics imbalance, disrupted cellular energy metabolism, oxidative stress, inflammatory responses, and alterations in Ca^2+^ homeostasis. To mitigate the risks of mitochondrial toxicity-induced cardiovascular diseases, innovative therapeutic strategies are being explored. These strategies aim to protect mitochondria by preserving their dynamics, enhancing endogenous ROS-scavenging mechanisms, restoring energy metabolism, or improving the clearance of damaged mitochondria [2]. Successful development of these approaches requires the implementation of advanced screening systems and well-characterized animal models to allow for efficient evaluation of their efficacy. Furthermore, a deeper understanding of the toxicity pathways in different treatment settings will be critical in identifying new therapeutic targets for tissue- and organ-specific toxicity. While many aspects of mitochondrial toxicity remain unclear, sustained research efforts promise significant advancements in patient protection from mitochondrial-related cardiac damage [110, 111]. Emerging targets and innovative approaches for addressing mitochondrial cardiac toxicity are explored in Table **6**, providing a snapshot of potential benefits and current research advancements.

### 7.1 Mitochondrial Biogenesis and Quality Control

Mitochondrial biogenesis and quality control are critical for maintaining a healthy mitochondrial network and ensuring proper cellular function. Biogenesis involves the synthesis of new mitochondria, which is regulated by factors such as intracellular energy levels, growth factor signaling, and cellular stressors. A healthy mitochondrial network is essential for maintaining energy production and cellular homeostasis. Mitochondrial quality control mechanisms-such as mitochondrial unfolded protein response, fission-fusion dynamics, mitophagy, and selective degradation-play a crucial role in preserving mitochondrial function by eliminating damaged organelles. Dysregulation of these processes can lead to cardiac diseases like ischemia-reperfusion injury, diabetic cardiomyopathy, and heart failure [118]. These mechanisms are central to ensuring the integrity of mitochondrial functions, and efforts to modulate these pathways could provide promising therapeutic avenues for treating mitochondrial dysfunctions in cardiac conditions [119, 120].

### 7.2 Gene Therapy and Mitochondrial Transplantation

Gene therapy offers significant promise for treating genetically inherited mitochondrial diseases. Emerging research has shown that targeted delivery of genetic material to cardiomyocytes can be achieved using phospholipid anchors with high targeting efficiency [121-123]. One such strategy involves gene therapy methods aimed at correcting mitochondrial genome defects using mitochondrial-targeted peptidyl-tRNA technologies, which have demonstrated success in animal models of mitochondrial disease and heart failure. Achieving adequate transgene expression, managing immunotoxicity, guaranteeing targeted tissue delivery, and creating efficient vectors are some of the difficulties in gene therapy. For gene therapy to be used successfully in the treatment of mitochondrial diseases, these barriers must be removed [124].

Mitochondrial transplantation, a potentially ground-breaking therapy, has gained attention as a treatment for diseases involving myocardial necrosis and loss of cardiomyocytes. Despite challenges like donor heart shortages and technical complexities associated with organ preservation, mitochondrial transplantation offers a viable alternative for improving heart function. Recent studies, including those by Zong *et al*., have provided proof-of-concept data showing the potential of delivering mitochon-drial preparations *via* transcutaneous or transvenous intracardiac approaches in animal models [125, 126]. The *ex vivo* perfusion methods used in cardiac transplantation could be adapted for the delivery of cell-free mitochondrial preparations, offering a promising solution to ischemic heart disease and other mitochondrial-related cardiac conditions [127-129]. Despite its potential, mitochondrial transplantation is still in the research phase. Potential immunological reactions, the long-term stability of the added organelles, and guaranteeing the survival and integration of transplanted mitochondria are among the difficulties. More research is required to completely understand and reduce these risks [130].

#### 7.2.1 Comparative Analysis

Novel strategies to address mitochondrial dysfunction in heart conditions are provided by gene therapy and mitochondrial transplantation. A mitochondrial transplant seeks to directly replace or supplement damaged mitochondria, whereas gene therapy focuses on repairing genetic defects to improve mitochondrial function. Although preclinical models have demonstrated the effectiveness of gene therapy, vector design and immune responses remain obstacles. Even though mitochondrial transplantation has shown promise in enhancing cardiac function, there are still unanswered questions about the longevity and integration of transplanted mitochondria. To ascertain both therapies' safety, effectiveness, and suitability for use in clinical settings, more research and clinical trials are required [124, 130].

Several issues need to be resolved to guarantee the safety and effectiveness of gene therapy and mitochondrial transplantation in the clinical treatment of mitochondrial dysfunction in cardiac disorders.

The clinical application of gene therapy and mitochondrial transplantation for treating mitochondrial dysfunction in cardiac diseases, several challenges must be addressed to ensure safety and efficacy.

#### 7.2.2 Gene Therapy: Gene Editing

##### 7.2.2.1 Challenges

CRISPR-Cas9 and other gene editing technologies have the ability to introduce unwanted changes at genomic locations that closely resemble the target sequence. These off-target edits could have negative consequences if they interfere with crucial genes or regulatory area [131]. A major obstacle still stands in the effective delivery of gene-editing components to particular cardiac cells. Poor delivery may lead to less-than-ideal editing and unintended consequences [132]. The immune system may react negatively to gene-editing components because it perceives them as alien, which could reduce the therapy's efficacy or result in inflammation [133].

#### 7.2.3 Mitochondrial Transplantation: Immune Rejection

##### 7.2.3.1 Challenges

Immune reactions may occur when exogenous mitochondria are introduced. According to studies, extracellular mitochondria can stimulate vascular endothelial cells, which can cause them to produce inflammatory cytokines and chemokines. This could potentially increase transplant rejection in models [134]. A lower risk of immunological rejection makes autologous mitochondrial transplantation using the patient's own mitochondria generally preferred. However, the process of extracting and preparing autologous mitochondria for transplantation presents both technical and logistical challenges [135]. It is essential to guarantee the transplanted mitochondria long-term survival and functional integration. Maintaining mitochondrial activity and avoiding rejection or degradation over time are challenges [136].

Techniques for reducing obstructions developing particular gene editing techniques in enhancing targeting precision and decreasing off-target effects can be achieved by creating sophisticated gene-editing instruments and delivery systems. Potential tactics include optimizing delivery vectors and using high-fidelity CRISPR-Cas9d variants [137].

Transplanting mitochondria through immune modulation. It may be possible to lessen immune rejection by using immunosuppressive techniques or designing mitochondria to avoid immune detection. The development of successful strategies requires more research [138]. For gene therapy and mitochondrial transplantation to be successfully used in clinical settings to treat mitochondrial dysfunction in cardiac diseases, these issues must be resolved.

## 8 SUMMARY OF KEY FINDINGS

Mitochondria are intracellular bioenergetic organelles that are responsible for ATP production. Mitochondria play a pivotal role in the regulation of redox, calcium homeostasis, apoptosis, necrosis, and autophagy. Mitochondria can exist as individual, filamentous, or tubular structures, depending on the metabolic and developmental status of living cells. Mitochondrial dynamics is a termine for the process of fission and fusion, which constantly change the size, shape, and number of mitochondria within the cells. Mitochondrial fission and fusion help to preserve the integrity of mitochondria, thereby maintaining normal mitochondrial size and morphology [139-142]. The dynamic behavior of mitochondrial networks is also known to respond to environmental signals and stressors. Disruption of normal mitochondrial dynamics has been implicated in the etiology of a variety of diseases, including cancer, neurodegenerative disorders, and cardiovascular diseases. An increase in mitochondrial fission would lead to the rapid fragmentation of mitochondria, whereas an increase in mitochondrial fusion would lead to mitochondrial elongation [2].

Despite the compartmentalization of excitation-contraction coupling, the rush of Ca^2+^ ions generated by the opening of the voltage-sensitive L-type Ca^2+^ channels is seen at the same time in both cell compartments, cytosolic and mitochondrial. The mitochondrial Ca^2+^-uptake is based on a proton (H)-independent Ca^2+^ uniport across the mitochondrial inner membrane (mIM) controlled by the electrochemical proton gradient H^+^in < H^+^out. This effect is mainly responsible for the steep increase of the intra-mitochondrial Ca^2+^ level [Ca^2+^]m from nanomolar to micromolar range within milliseconds of the onset of systolic Ca^2+^ rise. Mitochondrial Ca^2+^ is rapidly extruded by the Na^+^-dependent Ca^2+^ (Na^+^/Ca^2+^ exchangers- NCX) or H^+^-dependent Ca^2+^ export. The increase of the cationic level in mitochondria activates a large number of mitochondrial dehydrogenases, contributing to the high-energy phosphate production [143-146].

## 9 FUTURE RESEARCH DIRECTIONS

To gain a deeper understanding of the molecular mechanisms of mitochondria-related cardiac toxicity, further research should be conducted in the following areas. First, as abnormalities in mitochondria are validated in cardiotoxicity cases related to anticancer treatment or exposure to other toxic agents, new mitochondrial targets for therapeutic intervention focusing on cardioprotective strategies may be developed [147, 148]. This can alleviate the cardiac side effects of many cytotoxic chemotherapeutics while preserving their antitumor actions. Such approaches include enhancing intracellular defense mechanisms against oxidative stress, stabilizing mitochondrial bioenergetics, and promoting effective mitochondrial dynamics and quality control. Additionally, challenges in gene therapy for mitochondrial dysfunction need to be addressed. While gene therapy offers great promise, limitations such as efficient delivery systems, tissue-specific targeting, and potential immune responses must be overcome [149, 150].

Early detection of cardiac toxicity is crucial for timely intervention, particularly in patients receiving anthracyclines, as these often present with asymptomatic abnormalities before the onset of heart failure. Novel biomarkers—such as circulating cell-free DNA, microRNAs, and proteins released into circulation due to necrotic cell death—may be established to noninvasively assess mitochondrial and cardiac injury at its earliest stages. These advances may subsequently be improved to facilitate the screening of case screening populations that are sensitive to routine CVDs or experiencing myotoxicity from toxic drugs. Limitations in current diagnostic methods, particularly in detecting early-stage mitochondrial dysfunction, highlight the need for developing more sensitive and specific diagnostic tools. The integration of advanced imaging techniques, coupled with molecular markers, could enhance the accuracy of early mitochondrial dysfunction detection.

Cardiac dysfunction induced by anticancer agents is a clinically significant and pertinent research question for the future. Translational models (mice, rats, nonhuman primates) of various anticancer agents relevant to specific clinical scenarios may be developed to gain insight into the role of mitochondrial dysfunction in functional, structural, and metabolic disturbances in specific contexts (as in diabetes or aging). By improving understanding of the mechanisms by which toxic agents induce detrimental actions on heart mitochondria, new strategies and interventions to counteract this toxicity may be developed. Furthermore, exploring novel therapeutic targets such as mitochondrial biogenesis modulators and sirtuin activators could offer potential strategies for mitigating mitochondrial dysfunction and cardiac toxicity. These molecules have shown promise in preclinical studies and may provide novel avenues for drug development in this area [150-158].

## 10 ACKNOWLEDGING THE LIMITATIONS OF THE STUDY

Acknowledging the limitations in medical research concerning mitochondria and the heart reveals a scarcity of clinical data and technological advancements. Completed studies, whether small or large trials, rely on “unique” patient populations of “zero” size and methodologies with significant limitations. Differences in clinical trials arise from various approaches in patient selection and study design, introducing biases that must be considered when forming conclusions about the mechanisms linking mitochondrial dysfunction to heart failure development. This review explores potential biological mechanisms contributing to impaired signal transduction or bioenergetic dysfunction in the heart. Additional research is necessary to understand how mitochondrial dysfunction affects heart failure outcomes, as proposing interventions aimed at preserving mitochondrial integrity should be treated with caution. Moreover, standardizing the measurement of signal molecules or enzymatic activities across clinical centers poses a challenge. The review does not provide clear guidelines for managing patients with cardiac mitochondrial defects, although analyzing bioenergetics through standardized ATP production patterns and glycolytic enzyme sensitivity may be beneficial. However, this capability is limited to research centers with the necessary molecular biology facilities, which are not yet widespread in clinical or research settings [159-162].

## CONCLUSION

The heart is optimized for normal functioning by mitochondria, a fact that is attributed to their role in energy production, calcium equilibrium, and ROS management. Mitochondrial breakdown, however, plays a primary role in cardiac toxicity, especially in instances such as chemotherapy-induced cardiotoxicity and impairment of the heart’s mitochondria due to drugs. Within these mechanisms of this toxicity, ranging from damage to mitochondrial DNA to the alteration of the functioning of the mitochondrial permeability transition pore, these three elements of oxidative stress, bioenergetic compromise, and cell death form a vicious cycle.

Tachycardia, heart failure, or other clinical consequences of cardiac toxicity due to the compromised function of mitochondria call for the availability of relevant diagnostics, imaging or biomarkers around the heart injury. In most cases, treatment is associated with medications or lifestyle and nutrition changes, but rather few are available for eliminating the consequences of mitochondrial damage.

New forms of treatment like stimulation of biogenesis and quality control of mitochondria, along with novel techniques such as gene therapy and mitochondrial transplantation, are being developed to lessen drug-induced toxicity. However, much progress has been made, but there still exists a lacuna concerning the understanding of the exact molecular mechanisms involved and their effect in various clinical conditions.

In the next multiple years, the emphasis of research should be on the management and monitoring of biological systems, avoiding off-target effects during focused therapy, and developing implementation of precision medicine. These aspects of knowledge development have the promise of changing the prognosis in clinical practice and the life of patients in a positive way.

## Figures and Tables

**Fig. (1):**
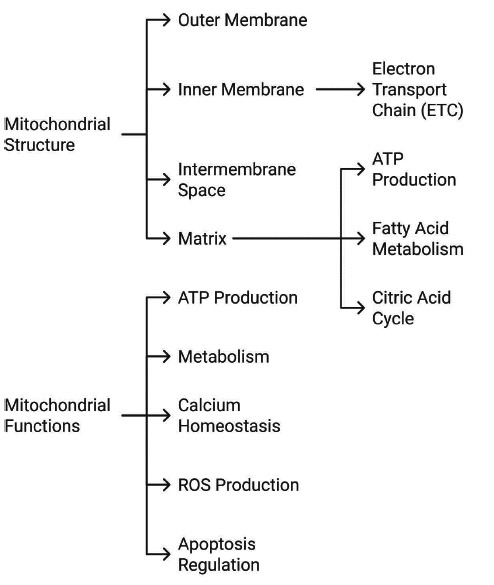
Structural components and functional roles of mitochondria.

**Fig. (2):**
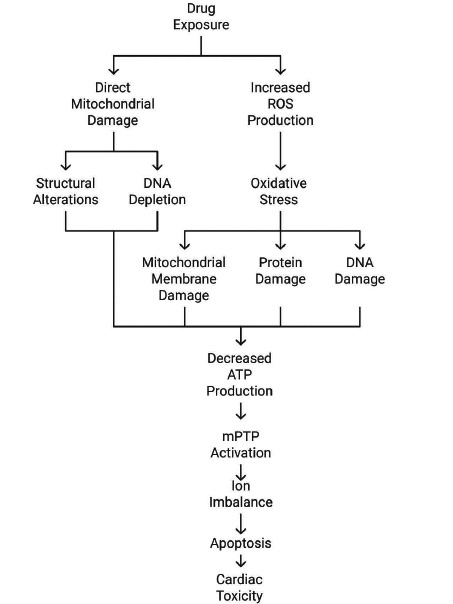
Mechanisms through which drug exposure induces mitochondrial dysfunction and subsequent cardiac toxicity.

**Fig. (3):**
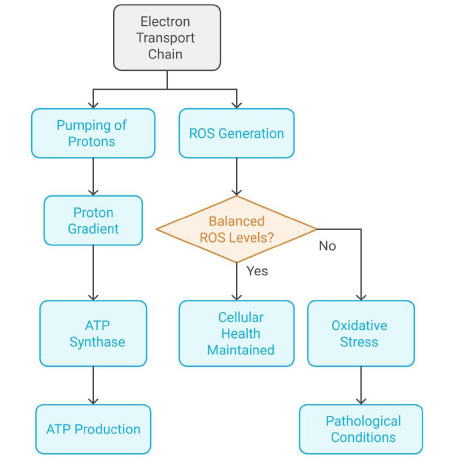
Dual role of the mitochondrial electron transport chain (ETC) in cellular metabolism and redox balance.

**Fig. (4):**
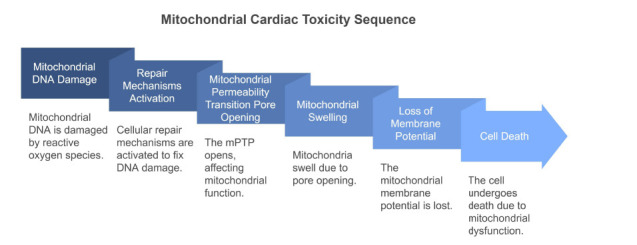
Sequence of events involved in mitochondrial cardiac toxicity.

**Fig. (5):**
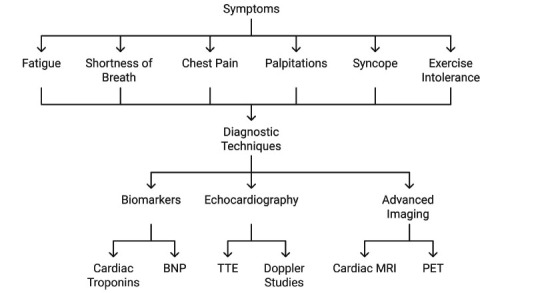
Overview of the symptoms and diagnostic techniques associated with cardiac conditions.

**Fig. (6):**
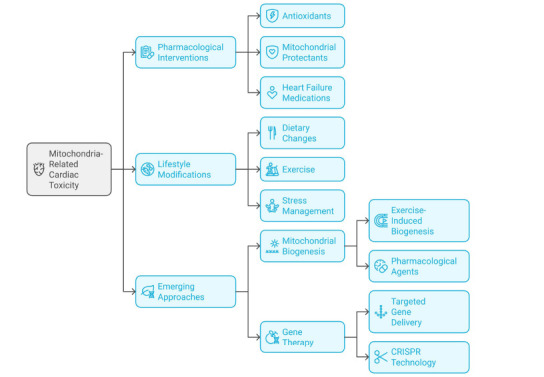
Various approaches to mitigate mitochondria-related cardiac toxicity.

**Table 1:** Summary of chemotherapy drugs and associated cardiotoxic effects.

**Drug Name**	**Mechanism of Cardiotoxicity**	**Mitochondrial Effects**	**Clinical Outcomes**	**References**
Doxorubicin	Inhibition of topoisomerase II leads to DNA damage	Mitochondrial DNA damage, disruption of oxidative phosphorylation	Cardiac dysfunction, heart failure, arrhythmias	[26]
Trastuzumab	HER2 receptor inhibition in cardiac cells	Impaired mitochondrial function, decreased ATP production	Left ventricular dysfunction, heart failure	[27]
Cyclophosphamide	DNA alkylation and cross-linking, leading to cell apoptosis	Increased oxidative stress, mitochondrial dysfunction	Dilated cardiomyopathy, heart failure	[28]
5-Fluorouracil	Inhibition of thymidylate synthase, inducing DNA damage	Mitochondrial damage through increased ROS production	Myocardial ischemia, arrhythmias	[29]
Bevacizumab	Vascular endothelial growth factor inhibition	Altered mitochondrial biogenesis and function	Hypertension, myocardial infarction	[30]
Anthracyclines (*e.g.*, Epirubicin)	DNA intercalation, inhibition of topoisomerase II	Increases in mitochondrial ROS, mitochondrial permeability transition pore opening	Left ventricular dysfunction, myocardial infarction, arrhythmias	[31]

**Table 2:** Key mitochondrial dysfunction markers in cardiac pathophysiology.

**Marker**	**Role in Mitochondrial Function**	**Relevance to Cardiac Toxicity**	**Detection Methods**	**References**
Cytochrome c	Involved in the electron transport chain and apoptosis regulation	Released into the cytosol during mitochondrial dysfunction, leading to apoptosis and cardiac cell death	Western blot, ELISA, Immunohistochemistry	[38]
Mitochondrial DNA (mtDNA)	Responsible for encoding proteins critical for mitochondrial function	mtDNA damage is linked to impaired mitochondrial function and cardiac toxicity	PCR-based assays, sequencing, qPCR	[39]
Mitochondrial Membrane Potential (Δψm)	Maintains the electrochemical gradient crucial for ATP synthesis	Depolarization of Δψm is a key indicator of mitochondrial dysfunction in cardiac toxicity	Flow cytometry, JC-1 assay	[40]
Reactive Oxygen Species (ROS)	Byproducts of mitochondrial oxidative phosphorylation	Excessive ROS contributes to oxidative stress and mitochondrial injury, exacerbating cardiac toxicity	Chemiluminescence, DCFDA assay, Mass spectrometry	[41]
Peroxisome Proliferator-Activated Receptor Gamma Coactivator 1α (PGC-1α)	Regulates mitochondrial biogenesis and energy metabolism	Reduced PGC-1α activity is associated with mitochondrial dysfunction and cardiomyopathy	Western blot, qPCR	[42]
Adenine Nucleotide Translocase (ANT)	Regulates ATP/ADP exchange across the mitochondrial inner membrane	ANT dysfunction is linked to impaired mitochondrial energy production and cardiac toxicity	Western blot, Immunofluorescence	[43]

**Table 3:** Mitochondrial DNA damage and repair pathways in cardiac toxicity.

**Type of Damage**	**Repair Mechanism**	**Impact on Cardiac Health**	**Clinical Relevance**	**References**
Mitochondrial DNA (mtDNA) lesions	Base excision repair (BER)	Accumulation of mtDNA damage leads to impaired oxidative phosphorylation and ATP production	Increased cardiac vulnerability to ischemia/reperfusion injury	[64]
mtDNA mutations	Mitochondrial DNA polymerase γ (POLG) repair	Genetic mutations disrupt mitochondrial function, causing arrhythmias and heart failure	POLG dysfunction is linked to dilated cardiomyopathy	[65]
Double-strand breaks	Homologous recombination, non-homologous end joining	Leads to mtDNA instability, contributing to oxidative stress and apoptosis	Mutations in repair proteins exacerbate chemotherapy-induced cardiac toxicity	[66]
mtDNA depletion	Mitochondrial DNA replication machinery repair	Reduced mitochondrial mass and energy production impair cardiomyocyte function	Mitochondrial depletion syndromes show severe cardiac manifestations	[67]
Aging-related mtDNA damage	Mitochondrial autophagy (mitophagy) and repair enzymes	Accumulation of damaged mtDNA accelerates aging-related cardiac dysfunction	Impaired mitophagy increases the risk of heart failure in elderly patients	[68]

**Table 4:** Diagnostic approaches for mitochondria-related cardiac toxicity.

**Technique**	**Target Biomarker/Imaging Focus**	**Advantages**	**Limitations**	**References**
Echocardiography	Cardiac function, Left ventricular ejection fraction (LVEF)	Non-invasive, Widely available, Real-time monitoring	Limited sensitivity in the early stages of toxicity	[77]
Magnetic Resonance Imaging (MRI)	Cardiac fibrosis, Myocardial viability	High-resolution images, Non-invasive, Quantitative measurement of fibrosis	Requires specialized equipment, High cost	[78]
Biomarkers (Troponin, BNP)	Troponin I, B-type natriuretic peptide (BNP)	Simple blood test, High specificity for myocardial injury	Not specific to mitochondrial toxicity, can be affected by other conditions	[79]
PET Imaging (18F-FDG)	Mitochondrial metabolic activity	Detects altered mitochondrial metabolism, a Quantitative measure	Limited resolution, requires specialized expertise	[80]
Mitochondrial DNA Quantification	Mitochondrial DNA copy number, Mitochondrial dysfunction markers	Early detection of mitochondrial damage, Direct assessment of mitochondrial health	Invasive in certain cases, requires specialized equipment	[81]
Optical Coherence Tomography (OCT)	Myocardial tissue structural changes	High spatial resolution, Non-invasive, Can visualize microvascular changes	Limited depth penetration, Sensitive to movement	[82]

**Table 5:** Pharmacological and lifestyle interventions for managing cardiac toxicity.

**Intervention Type**	**Mechanism of Action**	**Mitochondrial Target**	**Effectiveness**	**References**
ACE Inhibitors	Reduce cardiac stress and improve ventricular function	Mitochondrial oxidative stress	Effective in reducing heart failure and mitigating oxidative damage	[87]
Statins	Inhibit cholesterol synthesis, reduce ROS production	Mitochondrial ROS production	Statins are beneficial in mitigating mitochondrial dysfunction and improving heart function	[88]
Metformin	Improves mitochondrial function and enhances energy production	Mitochondrial respiration and ATP production	Effective in reducing oxidative stress and improving mitochondrial bioenergetics	[89]
Mitochondrial-targeted Antioxidants (*e.g.*, MitoQ)	Directly scavenge ROS and protect mitochondria	Mitochondria (electron transport chain)	Shown to protect against chemotherapy-induced cardiac toxicity	[90]
Exercise Training	Enhances mitochondrial biogenesis and function	Mitochondrial biogenesis	Regular exercise improves mitochondrial health and reduces cardiac dysfunction	[91]
Dietary Interventions (*e.g.*, Coenzyme Q10 supplementation)	Improves mitochondrial energy production and reduces oxidative stress	Mitochondrial electron transport chain	CoQ10 supplementation has been shown to reduce the impact of mitochondrial dysfunction in cardiac toxicity	[92]
Ppar-α Agonists (*e.g.*, Fenofibrate)	Activates genes involved in mitochondrial fatty acid oxidation	Mitochondrial fatty acid oxidation	Effective in improving cardiac function and mitochondrial health in cardiotoxicity	[93]

**Table 6:** Future therapeutic targets for mitochondrial cardiac toxicity.

**Target**	**Mechanism**	**Potential Benefits**	**Current Research Status**	**References**
Mitochondrial Biogenesis	Activation of pathways like PGC-1α to increase mitochondrial function and number.	Enhanced mitochondrial energy production, reduced ROS production.	Ongoing research into small molecules and gene therapies targeting PGC-1α and related pathways.	[112]
Mitochondrial Quality Control	Autophagic removal of damaged mitochondria *via* mitophagy.	Reduction of dysfunctional mitochondria, improved cardiac health.	Exploring mitophagy enhancers like urolithin A and their effects on heart health.	[113]
Mitochondrial Uncoupling Proteins (UCPs)	Regulation of proton gradient to reduce ROS and maintain cellular energy balance.	Reduced oxidative stress, protection against mitochondrial dysfunction.	Investigating the role of UCPs in cardiac protection in preclinical studies.	[114]
Mitochondrial Permeability Transition Pore (mPTP) Inhibitors	Blocking mPTP opening to prevent cell death following mitochondrial stress.	Prevention of mitochondrial swelling and apoptosis in cardiomyocytes.	Clinical trials investigating mPTP blockers like cyclosporine A and their cardioprotective effects.	[115]
Sirtuins (SIRT1)	Deacetylation of mitochondrial proteins to improve mitochondrial function and stress resistance.	Protection against cardiac damage from oxidative stress and mitochondrial dysfunction.	Clinical trials examining the effects of SIRT1 activators like resveratrol on heart failure.	[116]
Gene Therapy (Mitochondrial Gene Editing)	Correcting mutations in mitochondrial DNA to restore normal function.	Potential to treat inherited mitochondrial diseases and improve cardiac function.	Advances in CRISPR/Cas9 and other gene-editing technologies for mitochondrial DNA correction.	[117]

## LIST OF ABBREVIATIONS

ATP: Adenosine Triphosphate
CMs: Cardiomyocytes
ETC: Electron Transport Chain
IMM: Inner Mitochondrial Membrane
LS: Leigh Syndrome
ROS: Reactive Oxygen Species
